# Efficacy of parathyroid autotransplantation in endoscopic total thyroidectomy with CLND

**DOI:** 10.3389/fendo.2023.1193851

**Published:** 2023-06-27

**Authors:** Xiaozhou Cheng, Yaping Li, Lijun Chen

**Affiliations:** ^1^ Department of General Surgery, Gansu Provincial People’s Hospital, Lanzhou, China; ^2^ Department of Anesthesiology, Gansu Provincial People’s Hospital, Lanzhou, China; ^3^ Department of Radiology, Gansu Provincial People’s Hospital, Lanzhou, China

**Keywords:** endoscopic total thyroidectomy, central lymph node dissection, parathyroid transplantation, parathyroid hormone, hypoparathyroidism

## Abstract

**Purpose:**

To evaluate the safety and efficacy of autologous parathyroid transplantation in laparoscopic total thyroidectomy combined with central lymph node dissection (CLND).

**Methods:**

Retrospective analysis of clinical data of 152 patients admitted to the General Surgery Department of Gansu Provincial People’s Hospital who underwent endoscopic total thyroidectomy combined with CLND from June 2018 to December 2021. The intraoperative parathyroid glands were divided into the orthotopic preservation group (non-transplantation group) and the immediate active autologous transplantation group (transplantation group) according to the different treatment management of parathyroid glands during operation. The levels of Ca2+ in parathyroid blood and the incidence of hypoparathyroidism were compared between the two groups before operation and 1 day, 3 day, 1 week, 1 month, 3 months and 6 months after operation.

**Results:**

There was no significant difference in PTH between the preoperative transplantation group compared and the non-transplantation group (P>0.05); The PTH in the transplantation group was lower than that of the non-transplantation group 1 and 3 d after surgery, and the difference was statistically significant (P<0.05); No statistically significant difference in PTH between patients in the transplantation group compared with those in the non-transplantation group at 1 week postoperatively (P>0.05); PTH was significantly higher in the transplant group than in the non-transplant group at 1, 3 and 6 months after surgery, with statistically significant differences (P<0.05); there was no statistically differences (P>0.05) in serum Ca^2+^ between the preoperative, 1d, 3d and 1 week postoperative transplantation group and the non-transplantation group; Blood Ca^2+^ was significantly higher in the transplant group than in the non-transplant group at 1, 3 and 6 months after surgery, with statistically significant differences (P<0.05); The rate of temporary hypoparathyroidism in the transplantion group was higher than that in the non-transplantion group, and the rate of permanent hypoparathyroidism was lower than that in the non-transplantion group (P=0.044); There was no significant difference in the concentration of PTH in the cephalic vein of the elbow between the transplanted side and the non-transplanted side at 1 day and 3 days postoperatively (P>0.05); the concentration of PTH in cephalic vein of the elbow was significantly higher than that in non-transplanted side at 1 week, 1 month, 3 months and 6 months postoperatively (P<0.001); the number central area dissection and metastasis dissection in the transplantation group were significantly higher than those in the non-transplantation group (P<0.05).

**Conclusions:**

Most autologous parathyroid glands, having functional parathyroid autograft, is helpful to the occurrence of hypoparathyroidism after endoscopic total thyroidectomy with CLND, and it is an effective strategy to prevent permanent hypoparathyroidism, and more thorough area dissection is beneficial to the disease prognosis.

## 1 Introduction

The incidence of thyroid cancer is increasing yearly worldwide (1), and its most common pathological type is papillary thyroid cancer (PTC) (2). PTC is most prevalent in women, and surgery is the main treatment (3). However, traditional open surgery often uses a low collar incision, which will form a scar on the patient’s neck, affecting esthetics and causing a greater psychological burden on the patient’s work and life, especially for young and middle-aged women (4). In recent years, with the development of minimally invasive endoscopic surgery, and to better meet the cosmetic needs of patients, endoscopic surgery has been increasingly used worldwide in thyroid cancer surgery, with similar outcomes to those of traditional open surgery (5). The surgical approach is also being optimized from the previous cervical and axillary approaches to the current completely trans-areolar approach (6). Compared with open surgery, endoscopic total thyroidectomy reduces the size and improves the appearance of thyroid surgery incisions, satisfying the cosmetic needs of patients and reducing their psychological pressure. Simultaneously, the use of endoscopic magnification and endoscopic refinement of surgical operations results in a clearer surgical field, better identification and protection of important structures, such as the recurrent laryngeal nerve and parathyroid glands, fewer surgical complications, and improved quality of life (7). However, because of the close anatomical relationship between the parathyroid glands and the thyroid gland, the occurrence of postoperative hypoparathyroidism is difficult to completely avoid, regardless of the procedure used. Compared with simple thyroidectomy, patients undergoing total thyroidectomy combined with lymph node dissection in the central region are at a higher risk of parathyroid blood supply damage or miscutting, and the incidence of postoperative hypoparathyroidism is more significant. The literature reports that the incidence of transient hypoparathyroidism after thyroid surgery is 15%–30%, and the incidence of permanent hypoparathyroidism is 1%–3% (8). Transient hypoparathyroidism results in transient hypocalcemia. Permanent hypoparathyroidism causes localized numbness and tingling in the face and limb ends, and in severe cases, may be accompanied by hand and foot convulsions, and laryngeal and diaphragmatic spasms, which may be life-threatening. Calcium metabolism is also severely impaired. Some patients may be unable to work, and severe psychiatric symptoms may also occur. No artificial PTH preparation is currently available that can replace the function of the parathyroid glands. Parathyroid autotransplantation has been proposed as a technique to treat hypoparathyroidism (9). To further investigate the efficacy and safety of parathyroid autotransplantation in lumpectomy combined with lymph node dissection in the central region, this study selected patients with one inferior pole parathyroid gland that would be difficult to preserve *in situ* and who underwent immediate active autotransplantation. Postoperative serum parathyroid hormone level, blood calcium level, and the number of lymph nodes dissected in the central region were recorded, and postoperative efficacy was evaluated.

## 2 Materials and methods

### 2.1 Clinical information

The clinical data of 152 patients admitted to the Department of General Surgery of Gansu Provincial People’s Hospital from June 2018 to December 2021 for total lumpectomy of the thyroid combined with lymph node dissection in the central region were analyzed, and the case inclusion criteria were:(1) the patients were operated for the first time and the surgery was completed in our hospital;(2) the diagnosis of papillary thyroid cancer was confirmed or highly suspected by preoperative ultrasonography and fine needle aspiration, and the postoperative pathology confirmed the diagnosis of papillary thyroid cancer; (3) the preoperative parathyroid hormone levels and blood calcium were in the normal range; (4) the specific surgical procedure was chosen as lumpectomy with total bilateral thyroidectomy plus lymph node dissection in the central region. There were no statistically significant differences in age, gender, tumor size and stage, and surgical method between the two groups (P > 0.05), and the surgeries were performed by the same group of physicians. This study was approved by the Medical Ethics Committee of Gansu Provincial People’s Hospital(No.2022-195).

### 2.2 Surgical method

**Figure 1:**
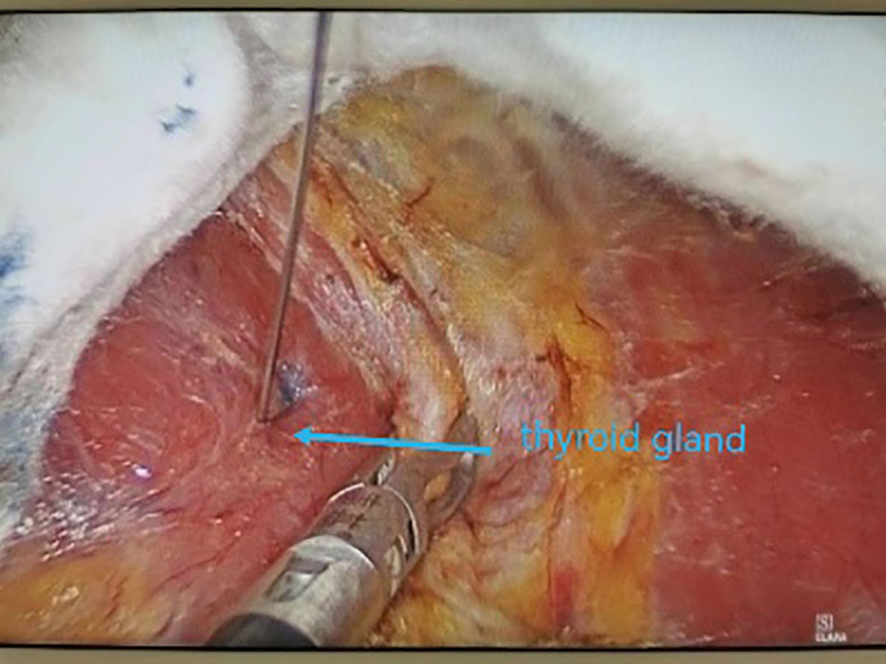
Percutaneous injection of nano-carbon.

**Figure 2:**
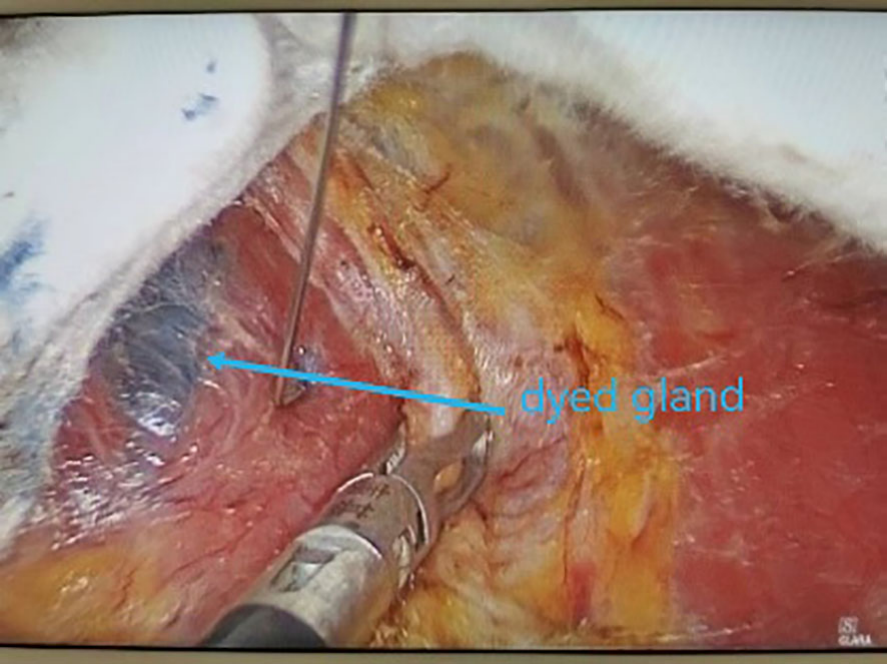
Dyed gland.

**Figure 3:**
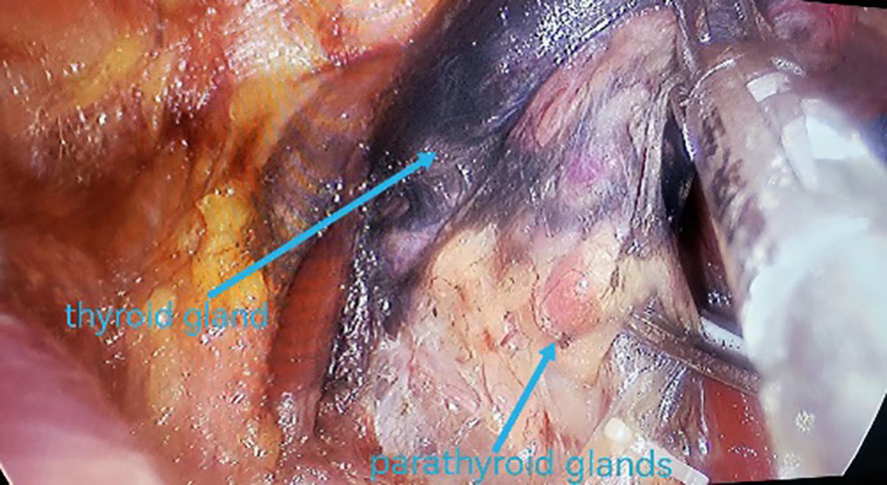
Parathyroid “negative opacification”.

**Figure 4:**
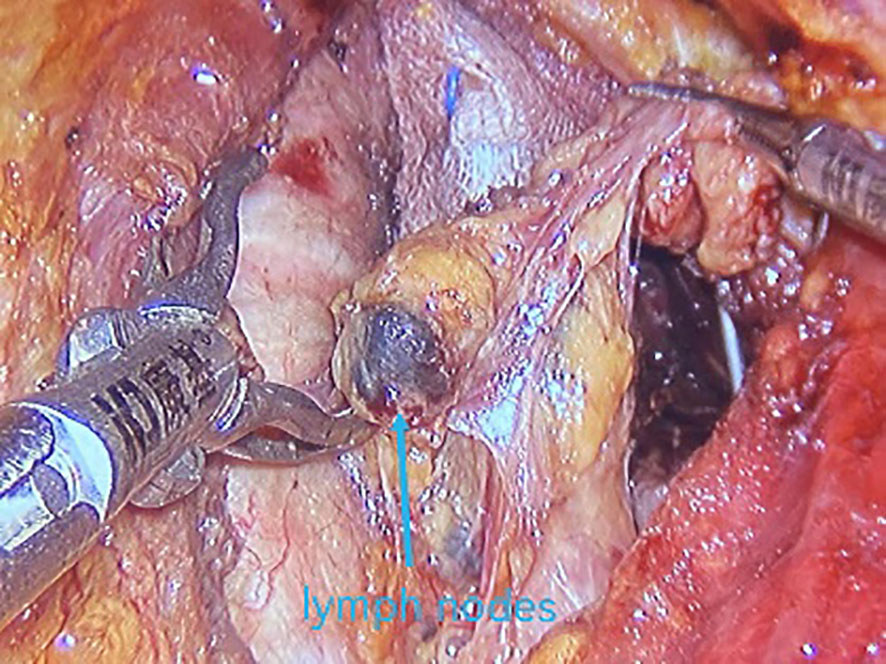
Stained lymph nodes.

**Figure 5:**
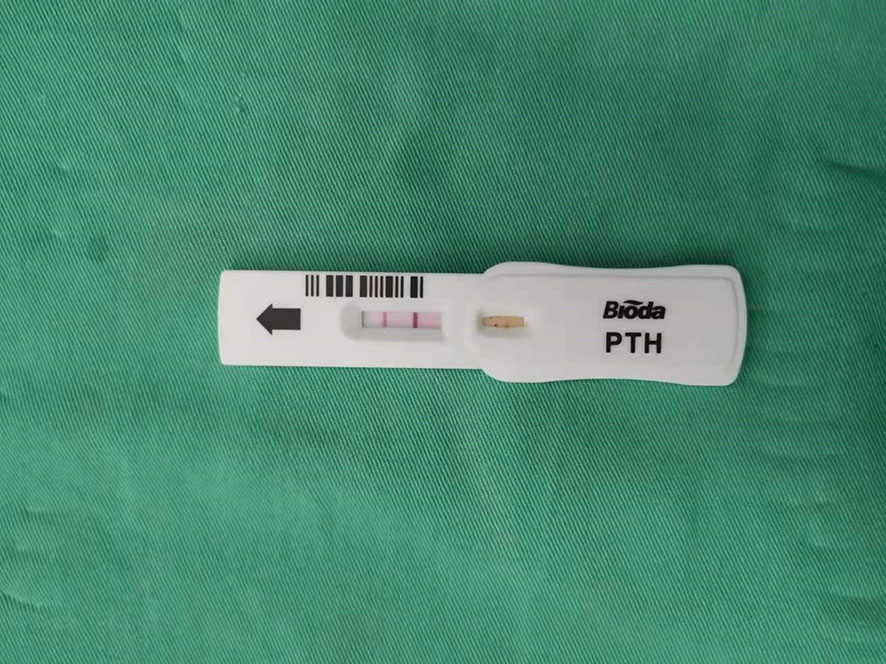
PTH test paper measurement.

**Figure 6:**
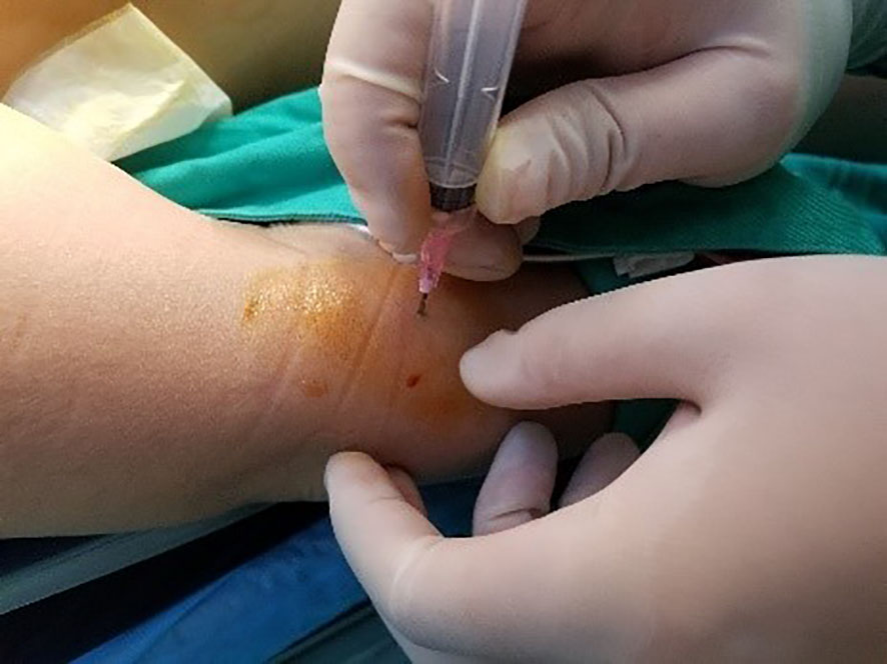
Brachioradialis homogenate injection.

All underwent general anesthesia and underwent lumpectomy for total bilateral thyroid excision plus lymph node dissection in the central region. Non-transplantation group: after revealing the thyroid gland, 0.2 ml of nanocarbon was extracted with a skin test needle, and 1~2 points were selected and injected into the gland by percutaneous puncture (**Figure 1**), about 0.1 ml per point, and the injection was backdrawn first to avoid accidental entry of the drug into the blood vessels, and the thyroid gland and lymph nodes were gradually blackened after about 10 min (**Figure 2**), followed by The operation was continued, and negative parathyroid image was seen (**Figure 3**), and all parathyroid glands were preserved in situ, and the black-stained lymph nodes in the central region were cleared (**Figure 4**). Transplantation group: For intraoperative parathyroid glands that were difficult to preserve in situ, the parathyroid glands were removed intact by blunt sharp combination using an ultrasonic knife and separating forceps, and placed in a specimen bag through the observation hole. After complete removal, the parathyroid glands were measured by PTH test paper (**Figure 5**), and then immediate active autotransplantation was performed by placing the isolated parathyroid glands immediately in 1 mL of 0.9% sodium chloride solution at 4 °C, cutting them into the smallest possible pieces with ophthalmic scissors to make a parathyroid suspension, and injecting them immediately into the forearm at the brachioradialis using a homogenous injection method radial (**Figure 6**). muscle using a homogenous injection method (**Figure 6**).

### 2.3 Observational indicators

(1) PTH and Ca^2+^ concentrations in the body circulation (non-transplanted forearm elbow head vein) were recorded before, 1 d, 3 d, 1 month, 3 months and 6 months after surgery, and the decision of whether to supplement calcium and the dose of calcium was made according to the patients’ blood Ca^2+^ status and clinical symptoms; (2) the occurrence of transient and permanent postoperative hypoparathyroidism was recorded; (3) the serum PTH concentrations in the elbow head vein were measured bilaterally at 1 d, 3 d, 1 month, 3 months and 6 months after surgery in the transplantation group; (4) the average number of lymph nodes detected in the central region was observed in both groups.

### 2.4 Statistical treatment

SPSS22.0 statistical software was used for analysis, and the measurement data were expressed as mean ± standard deviation (X ± s), and independent sample t-test was used for comparison between groups; the count data were expressed as rate (%), and χ2 test was used for comparison between groups, and the difference was considered statistically significant at P<0.05.

## 3 Results

### 3.1 General information

A total of 152 patients diagnosed with papillary thyroid cancer who underwent lumpectomy with total thyroid excision + lymph node dissection in the central region were included in this study. There were 80 cases in the transplantation group (immediate active autologous transplantation group) and 72 cases in the non-transplantation group (parathyroid gland preservation *in situ* group). Comparing the gender, age, preoperative PTH, and blood Ca^2+^ levels of the two groups, the differences were not statistically significant (P>0.05), as shown in **Table 1**.

**Table 1:** Baseline data of patients in the two groups.

Grouping	transplantation group	non-transplantation group	t/X^2^	P
Age	43.91 ± 11.33	42.96 ± 11.42	0.517	0.606
Gender(Male/female)	12/68	13/59	0.257	0.612
Preoperative PTH	58.90 ± 14.53	58.92 ± 13.17	-0.007	0.994
Preoperative Ca^2+^	2.330 ± 0.088	2.328 ± 0.090	0.119	0.905

### 3.2 Comparison of body circulation PTH and Ca^2+^ concentration after surgery between the two groups

Postoperative related indexes: 1d, 3 d and 1 week postoperatively, the circulating Ca^2+^ in the transplantation group was higher than that in the non-transplantation group, but the difference was not statistically significant (P>0.05); 1 month, 3 months and 6 months postoperatively, the circulating Ca^2+^ in the transplantation group was significantly higher than that in the non-transplantation group, and the difference was statistically significant (P<0.05); 1d and 3 d postoperatively, the circulating PTH in the transplantation group was lower than that in the non-transplantation group, and the difference was statistically significant (P<0.05); 1 week after surgery, systemic circulation PTH in the transplantation group was similar to that in the non-transplantation group, and the difference was not statistically significant (P>0.05), The systemic circulation PTH level in the transplant group was significantly higher than that in the non-transplant group 1 month, 3 months and 6 months after surgery, with statistical significance (P<0.05). The data are shown in **Table 2**.

**Table 2:** Comparison of Ca^2+^ and PTH concentrations in systemic circulation between the two groups.

Time	transplantation group	non-transplantation group	t/X^2^	P
n	80	72		
Ca^2+^				
Post 1 day	2.156 ± 0.113	2.138 ± 0.070	1.213	0.238
Post 3 day	2.162 ± 0.069	2.157 ± 0.074	0.376	0.707
Post 1 week	2.206 ± 0.064	2.192 ± 0.081	1.224	0.223
Post 1 month	2.260 ± 0.059	2.219 ± 0.067	4.030	0.000
Post 3 month	2.316 ± 0.060	2.274 ± 0.117	2.723	0.000
Post 6 month	2.318 ± 0.045	2.284 ± 0.094	2.815	0.000
PTH				
Post 1 day	15.58 ± 4.75	17.82 ± 6.01	-2.569	0.011
Post 3 day	18.48 ± 5.11	22.44 ± 7.36	-3.822	0.000
Post 1 week	24.68 ± 7.26	25.26 ± 7.46	-0.493	0.623
Post 1 month	47.84 ± 15.96	28.56 ± 9.50	9.155	0.000
Post 3 month	50.90 ± 14.72	30.79 ± 9.71	10.031	0.000
Post 6 month	51.89 ± 16.22	35.44 ± 12.26	7.090	0.000

### 3.3 Occurrence of hypoparathyroidism in both groups

A total of 31 cases (38.75%, 31/80) in the transplantation group had transient hypoparathyroidism and no permanent hypoparathyroidism; a total of 19 cases (26.39%, 19/72) in the non-transplantation group had hypoparathyroidism, of which 17 cases (23.61%, 17/72) had transient hypoparathyroidism and 2 cases (2.78%, 2/72) had permanent hypoparathyroidism. The incidence of transient hypoparathyroidism was higher in the transplantation group than in the non-transplantation group, and the incidence of permanent hypoparathyroidism was lower than in the non-transplantation group, with statistically significant differences (P<0.05) (**Table 3**).

**Table 3:** Occurrence of hypoparathyroidism in the two groups.

Grouping	THH	PHH	*X^2^*	*P*
transplantation group	31(31/80)	0	5.438	0.044
non-transplantation group	17(17/72)	2(2/72)		

THH, transient hypoparathyroidism; PHH, permanent hypoparathyroidism.

### 3.4 Comparison of PTH concentration in the elbow cephalic vein on the transplant side and the non-transplant side in transplant patients

There was no statistically significant difference between the PTH concentrations in the elbow cephalic vein on the grafted side and the non-grafted side at 1 d and 3 d postoperatively (P>0.05); the PTH concentrations in the elbow cephalic vein on the grafted side were significantly higher than those on the non-grafted side at 1 week, 1 month, 3 months, and 6 months postoperatively, and the difference was statistically significant (P<0.001). With the prolongation of the recovery time, the PTH concentrations on the grafted side and the non-grafted side showed an increasing trend at all postoperative time points, as shown in (**Table 4**).

**Table 4:** Comparison of PTH concentration in the cephalic vein of the elbow on the graft side and the non-graft side in the transplant group (pg/mL, $\bar{\text{X}}$ ±S).

Time	graft side	non- graft side	T	P
Post 1 day	15.98 ± 5.26	15.58 ± 4.75	0.505	0.614
Post 3 day	17.35 ± 5.95	18.48 ± 5.11	-1.283	0.201
Post 1 week	29.81 ± 7.98	24.68 ± 7.26	4.259	0.000
Post 1 month	77.84 ± 20.02	47.84 ± 15.96	10.480	0.000
Post 3 month	95.76 ± 22.21	50.90 ± 14.72	15.065	0.000
Post 6 month	102.26 ± 21.32	51.89 ± 16.22	16.817	0.000

### 3.5 Comparison of the number of lymph node metastases and the number of cleared lymph nodes in the central area between the two groups

The total number of lymph nodes cleared in the transplantation group was 726, with a mean of (9.08 ± 3.17) lymph nodes per case, while 566 lymph nodes were detected in the non-transplantation group, with a mean of (7.86 ± 2.45) lymph nodes per case, and the mean number of lymph nodes detected in the transplantation group was significantly higher than that in the non-transplantation group. The average number of metastatic lymph nodes detected in the transplantation group was 269, with a mean of (3.36 ± 1.71) per case, and 204 in the non-transplantation group, with a mean of (2.83 ± 1.51) per case, and the average number of metastatic lymph nodes detected in the transplantation group was significantly higher than that in the non-transplantation group, and the difference was statistically significant (t=2.014,P<0.05) (**Table 5**).

**Table 5:** Comparison of the number of central lymph node metastases and the number of dissection between the two groups.

Groupings	*n*	CLND	positive lymph nodes
Experimental group 80	80	9.08 ± 3.17	3.36 ± 1.71
Control group 72	72	7.86 ± 2.45	2.83 ± 1.51
T		2.657	2.014
P		0.009	0.046

## 4 Discussion

Normal parathyroid glands are located on the dorsal side of the thyroid gland and are brownish-yellow in color and small in size. Most people have four parathyroid glands, but approximately 15% of the Chinese population has two parathyroid glands (10).The location of the superior parathyroid glands is relatively fixed, with 85% of them concentrated within a radius of 1 cm around the inferior angle of the thyroid cartilage, while the location of the inferior parathyroid glands is more variable and can be located in any area of the inferior pole of the thyroid, thymus, or even the mediastinum. The parathyroid glands are classified into two types according to their relationship with the thyroid gland and the ease of *in situ* preservation: type A is the compact type, which means that there is a close relationship between the parathyroid glands and the thyroid gland, and *in situ* preservation is relatively difficult; type B is the non-compact type, which means that there is a natural gap between the parathyroid glands and the thyroid gland, and *in situ* preservation is relatively easy (11). This type is not dependent on the surgeon’s surgical skills, experience, or other personal factors. The anatomical relationship between the parathyroid glands and the thyroid gland can be objectively assessed, thus helping the surgeon to effectively determine whether the parathyroid glands should be preserved *in situ* or autologously transplanted. The blood supply to the parathyroid glands is relatively homogeneous, with 80% of the blood supply to the superior parathyroid glands coming from the superior branch of the inferior thyroid artery and the rest from branches of the superior thyroid artery or anastomosing branches of the superior and inferior thyroid arteries. The blood supply to the inferior parathyroid glands mainly comes from the inferior thyroid artery, and most are supplied by independent terminal arteries. The blood supply to the parathyroid glands should be protected intraoperatively. When preserving the parathyroid glands in situ, emphasis should be placed on protecting the blood supply and avoiding complete “nakedness” of the parathyroid glands. Because total thyroidectomy combined with lymph node dissection in the central region can easily destroy the 3-polar terminal vessels of the parathyroid glands, timely identification of the location of the parathyroid glands and determination of their blood supply are crucial to their protection. The use of a nano-carbon (12) negative contrast technique combined with visual identification helps to quickly identify the parathyroid glands. Owing to the fragility of the parathyroid blood supply, intraoperative reliance on parathyroid color changes to determine whether the blood supply is impaired is unreliable. Complete preservation of the entire parathyroid blood supply is difficult, and overdissected small vessels may form thrombi or lose secretory function due to glandular edema. Lang et al. (13) suggested that the incidence of permanent hypoparathyroidism is higher if no color change occurs in any of the four parathyroid glands explored intraoperatively, possibly because local ischemia of the parathyroid glands is more dangerous than venous congestion, and intraoperative parathyroid color may not change in local ischemia. One of the major complications of thyroidectomy is parathyroid gland injury, and postoperative hypoparathyroidism due to parathyroid injury has been a problem for thyroid surgeons. In a retrospective study of 386 patients, 20% of patients had their parathyroid glands mistakenly removed during thyroid surgery and were more likely to develop permanent hypoparathyroidism (14). As the extent of thyroid surgery increases, the chance of parathyroid damage increases and the risk of hypoparathyroidism increases. In total thyroidectomy, the rate of parathyroid miscutting is 5.1%–20% (15). One study (16) reported that transient and permanent hypoparathyroidism occurred in up to 51.9% and 16.2% of cases, respectively, after total thyroidectomy with total central zone lymph node dissection, compared with 36.1% and 7%, respectively, after total thyroidectomy with unilateral central zone lymph node dissection. In a meta-analysis that included 115 studies, the incidence of transient and permanent hypoparathyroidism after thyroidectomy ranged from 19% to 38% and 0% to 3%, respectively (17). Ozogul et al. (18) reported the incidence of permanent hypoparathyroidism after thyroidectomy to be 6.90%–49% and the incidence of transient hypoparathyroidism to be 0.4%–33%. Orloff et al. (19) defined permanent hypoparathyroidism as having postoperative serum PTH levels below the lower limit of laboratory reference values and hypocalcemia for more than 6 months. In this study, the reference time was 6 months after thyroid surgery; recovery from hypoparathyroidism within 6 months after surgery was defined as transient hypoparathyroidism, otherwise it was defined as permanent hypoparathyroidism. In a quality of life questionnaire survey of 252 patients with permanent hypoparathyroidism, despite regular calcium therapy, nearly two-thirds of patients felt that low calcium symptoms interfered with their work and life and their health was poor (20). How should the parathyroid glands be more properly managed while eradicating thyroid cancer? The literature (21) has reported that routine transplantation of at least one parathyroid gland at the time of total thyroidectomy is an effective way to prevent permanent hypoparathyroidism. In clinical practice, we believe that transplantation is the only option to restore the function of the accidentally removed gland. Parathyroid autotransplantation is the ideal way to obtain physiological blood calcium levels in hypoparathyroid patients, and this treatment plays an important role in reducing the occurrence of permanent hypoparathyroidism. In recent years, the use of PTH paper in parathyroid transplantation has made the intraoperative identification of parathyroid glands more efficient and rapid, thus allowing rapid transplantation of parathyroid glands that cannot be preserved *in situ* and improving the survival rate of transplanted tissue. The use of the PTH test paper method in parathyroid transplantation has been reported in the literature. In the literature, the sensitivity of the PTH test paper method for parathyroid determination was 97.4% with an accuracy of 98.3% (22).There are two views on parathyroid autotransplantation: one advocates selective autotransplantation and the other advocates conventional autotransplantation. Selective autotransplantation is the preferred choice of most surgeons, and is mainly used in cases of impaired parathyroid blood supply and accidental resection. However, selective autotransplantation is closely related to the surgeon’s experience and level of competence. Conventional autotransplantation refers to the removal of at least one parathyroid gland for autotransplantation during thyroid surgery. The prevailing view is that parathyroid autotransplantation is recommended only when parathyroid glands cannot be preserved *in situ* or are accidentally removed due to mechanical injury or impaired blood supply, and should not be routinely undertaken. Abboud et al. (23) routinely autotransplanted at least one parathyroid gland and routinely treated patients with calcium and vitamin D postoperatively and reported no occurrence of permanent hypoparathyroidism. Trupka et al. (24) performed routine autografting of at least one parathyroid gland during total thyroidectomy with a zero incidence of permanent hypoparathyroidism. Although routine autografting may prevent permanent hypoparathyroidism, it may increase the incidence of transient hypocalcemia (25). Some studies (26) have shown that there is a lower probability of permanent hypoparathyroidism in patients who have undergone parathyroid autotransplantation than in patients with *in situ* preserved parathyroid glands. Some studies (27) have also concluded that parathyroid autotransplantation does not prevent permanent hypoparathyroidism or even increases the risk of permanent hypoparathyroidism. However, the results of numerous studies have also shown that routine administration of one parathyroid autotransplantation can almost completely prevent severe permanent postoperative hypoparathyroidism (21). In this study, the incidence of transient hypoparathyroidism was higher in the transplantation group than in the non-transplantation group (38.75% vs. 23.61%); the rate of permanent hypoparathyroidism was lower in the transplantation group than in the non-transplantation group (0 vs. 2.78%), and the difference was statistically significant (P<0.05). Transient hypoparathyroidism occurred more commonly after transplantation, and there were no obvious symptoms of hypocalcemia such as general numbness and convulsions, and no permanent hypoparathyroidism occurred. The location of the parathyroid glands is generally within the fibrous capsule of the thyroid gland, and the arterial blood supply depends on the grade 3 vessels or capillaries of the thyroid artery, making it difficult to ensure a good blood supply during surgery. Venous blood flow is affected and some of the parathyroid glands become edematous, ischemic and necrotic, causing great uncertainty in parathyroid function, which may cause low parathyroid hormone levels and permanent hypoparathyroidism. For type A parathyroid glands that have been freed intraoperatively, are severely ischemic, or are difficult to preserve in situ, active autotransplantation is performed immediately upon identification. Active autotransplantation can shorten the operative time and requires identification of only one parathyroid gland, thus avoiding excessive dissection to preserve the parathyroid glands and facilitating better *in situ* preservation of the remaining parathyroid glands. At the same time, for patients at high risk of lymph node recurrence in the central region, a strategic parathyroid autotransplantation (1+x+1) protocol is recommended (28), in which at least one parathyroid gland with good blood supply is preserved *in situ* and one pathologically confirmed inferior parathyroid gland is routinely autotransplanted. This can effectively reduce the occurrence of permanent postoperative hypoparathyroidism and greatly improve surgical safety and thoroughness. To assess the blood supply of the parathyroid gland preserved in situ, the parathyroid envelope can be opened by needle pricking or shearing, or a small amount of the parathyroid gland can be cut to expose the parathyroid gland surface. If there is no obvious fresh blood leakage, then parathyroid autotransplantation should be performed immediately, rather than transplantation or preservation *in situ* without certainty.

Parathyroid autotransplantation methods include the granule embedding method and the homogenization method. In the granule embedding method, the parathyroid gland is cut into thin slices or granules <1 mm thick and scattered into a pocket separated from the sternocleidomastoid muscle on the opposite side of the lesion. In the homogenization injection method, the parathyroid gland tissue is cut to near homogenization, mixed with approximately 1 ml of 0.9% sodium chloride solution in a container and injected into the muscle. When using this method, the depth of the injection should be carefully considered and tapered off while injecting so that the graft is not too concentrated, which could affect survival. In the author’s opinion, the homogenization method of parathyroid tissue injection is simple, easy to implement, and effective. Moreover, the homogenization method can improve the survival rate of grafts and can more effectively avoid hypoparathyroidism after total lumpectomy of the thyroid gland. The reason for this may be that the tissue block in the homogenous injection method is smaller and more uniformly diffused, and the contact area with the muscle is larger, making it less prone to central ischemic necrosis and muscle hematoma. It is also more conducive to angiogenesis and more likely to survive, although the parathyroid tissue needs to be sufficiently clipped during transplantation. There are many different locations for parathyroid autografts, the common ones being the sternocleidomastoid, brachioradialis, pectoralis major, anterior cervical muscles, and the subcutaneous tissue of the forearm. An ideal autologous graft site should meet the following conditions: (1) high local partial pressure of oxygen, (2) high peripheral vascularization, and (3) ease of operation. Lo et al. (29) successfully demonstrated the function of transplanted parathyroid glands using the brachioradialis muscle as the graft site. In a lumpectomy for total thyroidectomy combined with central zone lymph node dissection, the anterior wall brachioradialis muscle was chosen as the immediate autograft site in this study to facilitate postoperative monitoring of graft function (venous blood parathyroid hormone on the grafted side versus the non-grafted side). The greatest advantage of this site is that it can be used as direct evidence of graft success by the gradient difference in parathyroid hormone measurements on the grafted side versus the non-grafted side, and it is easy to remove the graft.

Clinical assessment of postoperative parathyroid function after thyroid surgery is difficult, and can only be predicted by postoperative clinical symptoms, blood calcium levels, and PTH measurements, whether intraoperatively preserved *in situ* or following autologous transplantation. In this study, there were two secretory sites of PTH in the transplantation group (*in situ* parathyroid glands and transplantation site parathyroid glands), whereas there was only one secretory site in the non-transplantation group (*in situ* preserved parathyroid glands). In the first and third postoperative days, the PTH-secreting gland at the transplantation site did not have secretory function, and only the parathyroid gland *in situ* with good function secreted PTH in both groups, but the number of parathyroid glands *in situ* in the non-transplantation group was higher than that in the transplantation group, resulting in a statistically significant difference (P<0.05). The difference between the two groups was not statistically significant at 1 week after surgery (P>0.05), indicating that the parathyroid glands at the transplantation site had survived and started to secrete PTH within a week. At 1 month after surgery to 6 months after surgery, the circulating PTH in the transplantation group was significantly higher than that in the non-transplantation group, and the difference was statistically significant (P<0.05). The author believes that the transplantation group will be more favorable to the recovery of parathyroid function. However, the specific time for the graft to achieve normal PTH secretory function is more controversial. Some studies (29) showed that parathyroid tissue recovered function at 2–4 weeks after autologous transplantation, and fully recovered function after 8 weeks. El-Sharaky et al. (30) found that the transplanted parathyroid glands were ischemic and regressed at the first postoperative week, started to add value at the second week, and basically recovered normal secretory function at the fourth week. Cavallaro et al. (31) confirmed the survival of transplanted parathyroid glands by subcutaneous transplantation of parathyroid glands in the forearm and observed that serum parathyroid hormone levels were two to three times higher on the transplanted side than on the non-transplanted side. A prospective study compared postoperative parathyroid hormone concentrations in transplanted and non-transplanted forearms by injection at a new subcutaneous site close to the cephalic arm vein to provide a more precise method to confirm the success of parathyroid transplantation (32). The author believes that the simplest, most direct, and effective way to determine whether a transplanted parathyroid gland is viable is to determine whether PTH concentrations in the reflux venous blood at the transplanted parathyroid gland are higher than those in the venous blood of the body circulation; this would demonstrate the presence of a source of parathyroid hormone secretion at the transplantation site. The main cells of the parathyroid glands secrete parathyroid hormone, which is released into the blood in a cytosolic manner. If the transplanted parathyroid glands are viable and able to secrete PTH, then the PTH concentration in the cephalic vein of the elbow on the transplanted side must be higher than the PTH concentration in the body circulation. In this study, the cephalic vein on the opposite side of the graft was used as the blood sampling point for the body circulation). According to this principle, if the serum PTH concentration in the cephalic vein on the grafted side is greater than that on the contralateral side, and a certain difference is reached, the grafted parathyroid gland is proven to be functional. The results of this study showed that the difference in parathyroid hormone levels in the cephalic vein of both elbows at 1 and 3 days after surgery was small and the difference was not statistically significant, indicating that the transplanted parathyroid tissue takes time to become viable. The PTH concentrations in the elbow cephalic vein on the transplanted side were significantly higher than those on the non-transplanted side at 1 week, 1 month, 3 months and 6 months after surgery (P<0.001). The increase in the serum parathyroid hormone level was noticeable at the first week after surgery, indicating that the transplanted parathyroid tissue cells gradually became viable and partially resumed their secretory function. The serum parathyroid hormone level was significantly higher on the transplanted side than on the non-transplanted side at 1 month after surgery. Therefore, we believe that most parathyroid autografts resume normal secretory function by the fourth postoperative week. The recovery of parathyroid function may be related to the number of parathyroid glands damaged intraoperatively or accidentally removed, or to the function of parathyroid glands preserved *in situ* or autologously transplanted. Sitges-Serra et al. (33) suggested a “thyroid splinting” effect, suggesting that the postoperative recovery of the parathyroid glands is facilitated by high doses of calcium and osteopontin to maintain blood calcium in the normal–high range. In this study, the differences in Ca^2+^ in the body circulation between the two groups of patients at 1 d, 3 d and 1 week after surgery were not statistically significant (p>0.05). However, the Ca^2+^ concentration in the transplantation group was significantly higher than that in the non-transplantation group from 1 month to 6 months after surgery (p<0.05), showing that the Ca^2+^ concentration in the body circulation after surgery was not synchronized with the fluctuation of PTH, and there was a separation between the two. On the one hand, the author’s team routinely gave calcium and osteotriol to patients after surgery, which led to a certain bias in postoperative body circulation of Ca^2+^; on the other hand, there was a certain time difference in the mechanism of Ca^2+^ regulation by PTH. In parathyroid autotransplantation patients, the outcome of Ca^2+^ regulation by the parathyroid glands is multifactorial, and further experimental evidence is needed for the specific physiological process.

Lymph node dissection in the central region of thyroid cancer patients is an important risk factor for postoperative hypoparathyroidism. Therefore, protection of parathyroid gland function should be considered to prevent postoperative hypoparathyroidism during the process of central region lymph node dissection. Ligation or injury to the main trunk of the inferior parathyroid artery and the lingual lobe of the thymus should be avoided while ensuring complete dissection. Maximizing the blood supply to the parathyroid glands during surgery can effectively reduce the incidence of permanent postoperative hypoparathyroidism, but this undoubtedly increases the difficulty of surgery and the risk of incomplete lymph node dissection, especially in patients with anatomical variations of the parathyroid glands, which may increase the possibility and extent of injury if preserved *in situ* without distinction. To reduce the incidence of postoperative complications, incomplete “strawberry picking” lymph node dissection in the central region is sometimes performed, which may be an important cause of lymph node recurrence in the central region. Wei et al. (34) reported that transplantation of inferior parathyroid glands when central zone lymph node dissection was performed reduced not only the incidence of permanent postoperative hypoparathyroidism but also the risk of tumor recurrence. In the present study, 152 patients underwent lymph node dissection in the central region, and the average number of lymph nodes dissected per case was 9.08 ± 3.17 in the transplantation group and 7.86 ± 2.45 in the non-transplantation group. The average number of lymph nodes detected per case was significantly higher in the transplantation group than in the non-transplantation group (t=2.657, P<0.05). The average number of metastatic lymph nodes detected per case was 3.36 ± 1.71 in the transplantation group and 2.83 ± 1.51 in the non-transplantation group, and was significantly higher in the transplantation group than in the non-transplantation group (t=2.014, P<0.05). In the non-transplantation group, lymph node dissection in the central region was more conservative due to the protection of the inferior parathyroid glands, and the scope of surgery and the number of lymph nodes dissected were limited. In contrast, in the transplantation group, the number of lymph nodes cleared in the central region was higher than that in the non-transplantation group because the inferior parathyroid glands had been transplanted and the operator was not concerned about accidental dissection or damage to the blood supply of the parathyroid glands in the region, and thus more thorough and aggressive lymph node clearance could be performed. The number of positive lymph nodes was also higher in the transplant group than in the non-transplant group because the overall number of lymph nodes cleared was higher in the experimental group than in the non-transplant group. This result shows that active parathyroid autotransplantation does improve the completeness of lymph node clearance and greatly reduces the constraints on clinicians due to parathyroid protection, thus reducing the potential for postoperative lymph node recurrence. Therefore, when performing lymph node dissection in the central region, we should follow the “all or nothing” principle of regional dissection and try to protect the parathyroid glands and the small vessels supplying the parathyroid glands in situ. Lymph nodes to avoid local recurrence due to incomplete debridement.

There are some limitations in this study. During thyroid surgery, each step of the procedure was followed with refinement, and each parathyroid gland was carefully identified and each parathyroid gland found intraoperatively was protected as the last 1. However, this study lacks data on how many parathyroid glands were identified and preserved in both groups of patients. Unfortunately, this observation was not set as a key observation during the study, and detailed data were not recorded on the parathyroid glands identified and protected in both groups. In addition, the efficiency of autologous transplantation may also be related to the transplantation method and individual condition, which needs to be further studied to explore better methods to further improve the efficiency of parathyroid autologous transplantation and to better avoid the occurrence of permanent hypoparathyroidism.

Currently, the prevention of hypoparathyroidism is still based on the option of parathyroid preservation *in situ* and autologous transplantation. This study demonstrates that most autografted parathyroid glands are secretory and that immediate active autografting of parathyroid glands can help reduce the incidence of hypoparathyroidism after endoscopic total thyroidectomy with CLND, is an effective strategy to prevent permanent hypoparathyroidism, and allows for more complete lymph node dissection in the central zone to facilitate disease regression. The efficiency of autologous transplantation may also be related to factors such as transplantation method and individual conditions; these factors need to be further investigated to improve the efficiency of parathyroid autologous transplantation and to avoid permanent hypoparathyroidism. The authors suggest that a multicenter prospective randomized controlled clinical study of parathyroid autotransplantation should be conducted by a domestic thyroid surgery team, and that in-depth studies on the number of parathyroid autotransplants, graft sites, and molecular mechanisms be conducted to provide better quality clinical evidence on the effectiveness and safety of intraoperative parathyroid autotransplantation. In thyroid surgery, meticulous surgical technique is fundamental, and each parathyroid gland should be carefully identified and protected. More research is needed on the safety and efficacy of routine autologous transplantation of at least one parathyroid gland; however, most importantly, parathyroid glands that are not certain to survive should be transplanted, to avoid leakage, to prevent delayed hypoparathyroidism, and to avoid the occurrence of permanent hypoparathyroidism when possible. Many studies and the findings of practical clinical experience also favor parathyroid autotransplantation. Parathyroid autotransplantation techniques need to be standardized and more refined to continuously optimize autotransplantation protocols and improve transplantation success rates. This will ensure maximum benefit for patients undergoing thyroid surgery.

## Data availability statement

The original contributions presented in the study are included in the article/**Supplementary Material**. Further inquiries can be directed to the corresponding author.

## Ethics statement

The studies involving human participants were reviewed and approved by Medical Ethics Committee e of Gansu Provincial People’s Hospital (No.2022-195). The patients/participants provided their written informed consent to participate in this study. Written informed consent was obtained from the individual(s) for the publication of any potentially identifiable images or data included in this article.

## Author contributions

Conception and design: XC and LC. Administrative support: LC. Provision of study materials or patients: XC and YL. Collection and assembly of data: YL. Data analysis and interpretation: XC. Manuscript writing: all authors. Final approval of manuscript: all authors.
